# Arcanolysin is a cholesterol-dependent cytolysin of the human pathogen *Arcanobacterium haemolyticum*

**DOI:** 10.1186/1471-2180-11-239

**Published:** 2011-10-26

**Authors:** B Helen Jost, Erynn A Lucas, Stephen J Billington, Adam J Ratner, David J McGee

**Affiliations:** 1 Department of Veterinary Science and Microbiology, The University of Arizona, 1117 E Lowell Street, Tucson, AZ 85721, USA; 2 Ventana Medical Systems, Inc., 1910 Innovation Park Drive, Oro Valley, AZ 85755, USA; 3 Columbia University, Department of Pediatrics and Microbiology & Immunology, 650 W 168th Street BB443, New York, NY 10032, USA; 4 Louisiana State University Health Sciences Center-Shreveport, Department of Microbiology & Immunology, 1501 Kings Highway, Shreveport, LA 71130, USA

## Abstract

**Background:**

*Arcanobacterium haemolyticum *is an emerging human pathogen that causes pharyngitis, wound infections, and a variety of occasional invasive diseases. Since its initial discovery in 1946, this Gram positive organism has been known to have hemolytic activity, yet no hemolysin has been previously reported. *A. haemolyticum *also displays variable hemolytic activity on laboratory blood agar that is dependent upon which species the blood is derived.

**Results:**

Here we describe a cholesterol-dependent cytolysin (CDC) secreted by *A. haemolyticum*, designated arcanolysin (*aln*), which is present in all strains (n = 52) tested by DNA dot hybridization. Among the known CDCs, ALN is most closely related to pyolysin (PLO) from *Trueperella *(formerly *Arcanobacterium*) *pyogenes*. The *aln *probe, however, did not hybridize to DNA from *T. pyogenes*. The *aln *open reading frame has a lower mol %G+C (46.7%) than the rest of the *A. haemolyticum *genome (53.1%) and is flanked by two tRNA genes, consistent with probable acquisition by horizontal transfer. The ALN protein (~ 64 kDa) contains a predicted signal sequence, a putative PEST sequence, and a variant undecapeptide within domain 4, which is typically important for function of the toxins. The gene encoding ALN was cloned and expressed in *Escherichia coli *as a functional recombinant toxin. Recombinant ALN had hemolytic activity on erythrocytes and cytolytic activity on cultured cells from human, rabbit, pig and horse origins but was poorly active on ovine, bovine, murine, and canine cells. ALN was less sensitive to inhibition by free cholesterol than perfringolysin O, consistent with the presence of the variant undecapeptide.

**Conclusions:**

ALN is a newly identified CDC with hemolytic activity and unique properties in the CDC family and may be a virulence determinant for *A. haemolyticum*.

## Background

*Arcanobacterium haemolyticum*, a Gram positive, pleomorphic rod, causes wound infections and pharyngitis and can occasionally cause more severe invasive diseases such as endocarditis, meningitis, septic arthritis, pneumonia and osteomyelitis in humans [1]. There is strong epidemiologic evidence for *A. haemolyticum *being the only or primary isolate from throat specimens of some humans with pharyngitis [1-4] and these data suggest that the number of cases per year of *A. haemolyticum*-mediated pharyngitis is ~240,000-480,000 with 0.5-1 million lost work days in the United States. The organism, previously in the *Corynebacterium *genus, was classified as the first member of the genus *Arcanobacterium *[5]. The other members of the genus are uncommonly isolated and remain largely uncharacterized, with the exception of *Trueperella *(*Arcanobacterium*) *pyogenes*, which is an important opportunistic livestock pathogen [6].

Little is known about *A. haemolyticum *virulence factors with the exception of a phospholipase D (PLD) [7], which causes dermonecrosis [8]. We recently described the ability of PLD to reorganize host membrane lipid rafts, leading to enhanced bacterial adhesion [9]. Furthermore, *A. haemolyticum *was able to invade HeLa cells and once intracellular, PLD was able to kill host cells via direct necrosis [9]. These effects could potentially lead to bacterial dissemination to deeper tissues.

It is thought that clinical microbiology laboratories often miss *A. haemolyticum *in clinical specimens due to the organism's weak hemolytic activity on the commonly-used sheep blood agar, and therefore it may be misinterpreted as commensal diphtheroids and the isolate discarded. However, this organism displays more pronounced hemolysis on human and rabbit blood [10,11]. The organism has been known to have hemolytic activity since its initial discovery in 1946 [12], yet no *bona fide *hemolysin has been previously reported. PLD itself is not directly hemolytic, but causes synergistic hemolysis with bacteria that express cholesterol oxidase [13], prompting a search for the *A. haemolyticum *hemolysin. Possible clues to the identity of the *A. haemolyticum *hemolysin come from studies on the hemolytic bacterium *T. pyogenes*, which is closely related to *A. haemolyticum*. *T. pyogenes *expresses PLO, a member of the cholesterol-dependent cytolysin (CDC) toxin family, as its primary virulence factor and this molecule is a hemolysin [14]. Thus, we hypothesized that the hemolytic activity expressed by *A. haemolyticum *was due to the presence of an uncharacterized CDC.

Here we report the identification and characterization of a CDC from *A. haemolyticum*, designated arcanolysin (ALN). We show that ALN has several distinct structural features among the CDC family and demonstrate that ALN is cholesterol-dependent and provide evidence that ALN has variable hemolytic and cytotoxic activity against mammalian cells from different species. We propose ALN is the long, sought-after hemolysin.

## Methods

### Bacteria and growth conditions

ATCC 9345 is the *A haemolyticum *type strain. The other *A. haemolyticum *strains used in this study were archival isolates obtained from diverse human clinical cases (Table 1). *A. haemolyticum *and *Escherichia coli *strains were grown as previously described [9].

**Table 1:** *Arcanobacterium *strains used in this study.

Strain (all *A. haemolyticum *except as noted)	Relevant characteristics	Source
AhS1	Biotype S*; wound infection; 73 year old male; 1991	Petteri Carlson

AhS2	Biotype S; paronychia; 16 year old male; 1991	Petteri Carlson

AhS3	Biotype S; wound infection; 11 year old male; 1991	Petteri Carlson

AhS4	Biotype S; infected leg ulcer; 47 year old male; 1991	Petteri Carlson

AhS5	Biotype S; wound infection; 64 year old male; 1991	Petteri Carlson

AhS6	Biotype S; wound infection; 43 year old male; 1991	Petteri Carlson

AhS7	Biotype S; infected leg ulcer; 68 year old female; 1991	Petteri Carlson

AhS8	Biotype S; wound infection; 62 year old male; 1991	Petteri Carlson

AhS9	Biotype S; wound infection; 38 year old male; 1991	Petteri Carlson

AhS10	Biotype S; paronychia; 21 year old male; 1991	Petteri Carlson

AhS11	Biotype S; pharyngitis; 3 year old male; 1991	Petteri Carlson

AhS12	Biotype S; pharyngitis; 23 year old female; 1992	Petteri Carlson

AhS13	Biotype S; pharyngitis; 28 year old female; 1992	Petteri Carlson

AhS14	Biotype S; pharyngitis; 23 year old female; 1992	Petteri Carlson

AhS15	Biotype S; pharyngitis; 20 year old male; 1992	Petteri Carlson

AhS16	Biotype S; sinusitis; 41 year old male; 1990	Petteri Carlson

AhS17	Biotype S; sinusitis; 65 year old female; 1991	Petteri Carlson

AhS18	Biotype S; pharyngitis; 12 year old male; 1992	Petteri Carlson

AhS19	Biotype S; pharyngitis; 20 year old female; 1992	Petteri Carlson

AhS20	Biotype S; pharyngitis; 34 year old male; 1992	Petteri Carlson

AhS21	Biotype S; peritonsillar abscess; 15 year old male; 1996	Petteri Carlson

AhS22	Biotype S; pharyngitis, pneumonia; 42 year old male; 1996	Petteri Carlson

AhS23	Biotype S; diabetic foot gangrene; 45 year old male; 1997	Petteri Carlson

AhS24	Biotype S; tonsillitis; 16 year old female; 1998	Petteri Carlson

AhS25	Biotype S; metatarsal osteitis; 37 year old male; 1998	Petteri Carlson

AhR26	Biotype R; wound infection; 43 year old male; 1991	Petteri Carlson

AhR27	Biotype R; wound infection; 53 year old male; 1991	Petteri Carlson

AhR28	Biotype R; pharyngitis; 13 year old female; 1991	Petteri Carlson

AhR29	Biotype R (uncertain); peritonsillar abscess; 18 year old male; 1991	Petteri Carlson

AhR30	Biotype R; sinusitis; 14 year old male; 1992	Petteri Carlson

AhR31	Biotype R; peritonsillar abscess; 21 year old male; 1986	Petteri Carlson

AhR32	Biotype R; peritonsillar abscess; 15 year old female; 1992	Petteri Carlson

AhR33	Biotype R; pharyngitis; 26 year old male; 1992	Petteri Carlson

AhR34	Biotype R; pharyngitis; 15 year old male; 1992	Petteri Carlson

AhR35	Biotype R; pharyngitis; 18 year old male; 1992	Petteri Carlson

AhR36	Biotype R; pharyngitis; 21 year old male; 1992	Petteri Carlson

AhR37	Biotype R; peritonsillar abscess; 15 year old female; 1992	Petteri Carlson

AhR38	Biotype R; wound infection; 21 year old female; 1992	Petteri Carlson

AhR39	Biotype R; pharyngitis; 18 year old female; 1992	Petteri Carlson

AhR40	Biotype R; pharyngitis; 17 year old male; 1992	Petteri Carlson

AhR41	Biotype R; pharyngitis; 24 year old male; 1992	Petteri Carlson

AhR42	Biotype R; pharyngitis; 16 year old female; 1992	Petteri Carlson

AhR43	Biotype R; pharyngitis; 12 year old male; 1992	Petteri Carlson

AhR44	Biotype R; pharyngitis; 18 year old male; 1992	Petteri Carlson

AhR45	Biotype R; pharyngitis; 16 year old male; 1992	Petteri Carlson

AhR46	Biotype R; pharyngitis; 14 year old female; 1992	Petteri Carlson

AhR47	Biotype R; pharyngitis; 13 year old female; 1991	Petteri Carlson

AhR48	Biotype R; pharyngitis; 15 year old male; 1992	Petteri Carlson

AhR49	Biotype R; pharyngitis; 20 year old male; 1991	Petteri Carlson

AhR50	Biotype R; pharyngitis; 19 year old female; 1992	Petteri Carlson

CCUG39796	Biotype unknown; infected leg ulcer; 52 year old male; 1998	Petteri Carlson

ATCC9345; DSM 20595	Biotype unknown, 1946	American Type Culture Collection; [12]

*Trueperella (Arcanobacterium*) *pyogenes *BBR1	*plo nanH nanP cbpA fimAB tet*(W); Isolated from a bovine abscess	[14]

* S, Smooth type; R, rough type.

### DNA techniques

*E. coli *DH5αMCR plasmid DNA extraction, transformation, DNA restriction, ligation and agarose gel electrophoresis were by standard methods [15]. DNA hybridization was performed using the DIG DNA Labeling and Detection Kit (Roche). PCR DNA amplification was performed using Vent DNA polymerase (NEB) for 35 cycles of 1 min at 94°C, 1 min at 50°C and 1 min/kb at 72°C, with a final extension step of 72°C for 7 min.

### Nucleotide sequence determination and analysis

Prior to the recent GenBank deposit of the 1.986 MB genome from strain ATCC9345 (= DSM20595 = 11018) [16], we sequenced the same strain to > 20× coverage (454 Life Sciences), with ~1.945 MB of unique sequence (> 98% complete) with essentially identical sequence data. A translated ORF with amino acid similarity to CDCs, Arch_1062, was identified within this sequence. Oligonucleotide primers flanking this ORF were used to amplify the region by PCR. The nucleotide sequence was confirmed by automated DNA sequencing of both strands. The *aln *sequence data and flanking regions were submitted to the GenBank/EMBL/DDBJ databases under accession number FJ785427.

Database searches were performed using the BlastX and BlastP algorithms [17]. tRNA sequences were identified using the tRNAscan-SE program [18]. Signal sequence prediction was performed using SignalP [19]. Transcriptional terminators were identified using mfold [20]. Multiple sequence alignments were performed using CLUSTAL W [21], and tree construction was with the neighbor-joining algorithm and midpoint rooting, carried out in MacVector version 12.0.3 (MacVector, Inc.). PEST sequence prediction used the pestfind algorithm http://emboss.bioinformatics.nl/cgi-bin/emboss/epestfind.

### Cloning and purification of a recombinant, 6xHis tagged-ALN (His-ALN)

The *aln *gene, without the signal sequence, was amplified from *A. haemolyticum *ATCC9345 genomic DNA by PCR with His-ALNF (5'-CCCGGCGTTGCGGATCCAGTTGACGC-3') and ALN5 (5'-GGACCTTCTCGAGTATGTATCACTC-3') encoding *Bam*HI and *Xho*I sites (underlined in the primer sequence), respectively. These primers amplified a 1,669 bp product. The PCR fragment was digested with *Bam*HI-*Xho*I and cloned into pTrcHisB (Invitrogen), to generate pBJ51, which encoded the 63.7 kDa His-ALN. The final His-ALN translational fusion protein thus has the MWVGSQKHYFFYQDRGKIMTRRFLATVAGTALLAGAFAPGVAFG signal sequence removed and replaced with the sequence from the vector that leads to MGGSHHHHHHGMASMTGGQQMGRDLYDDDDKDP (6 His underlined). No other ALN native amino acids were removed. Cultures for purification of His-ALN were grown and lysed as described [9]. His-ALN was purified from the soluble cell fraction using TALON Metal Affinity Resin, as described (Clontech). His-ALN was eluted from the resin with 50 mM imidazole, 20 mM Tris-HCl, 100 mM NaCl, pH 8.0 (elution buffer). Total protein concentration was determined using Bradford Protein Assay Reagent (Bio-Rad).

For some experiments ALN was amplified from ATCC 9345 DNA using the primers ALN26-F (GCCGCCGCTAGCGTTGACGCTTCAACACAAACCGATCC) and ALN-R (GCCGCCCTCGAGTCACTCGCTATGAACGATGTTCTTG), cloned into expression vector pET28a (Novagen) using *Nhe*I and *Xho*I sites (underlined), and confirmed by sequencing. The *plo *gene encoding PLO was amplified from *T. pyogenes *ATCC 49698 DNA using the primers PYO28-F (GCCGCCCATATGGCCGGATTGGGAAACAGTTCG) and PYO-R (GCCGCCCTCGAGCTAGGATTTGACATTTTCCTC), cloned into pET28a using *Nde*I and *Xho*I sites (underlined), and confirmed by sequencing. The *ily *gene encoding ILY was amplified from *Streptococcus intermedius *and cloned into pET28a as described [22]. Purification of the His-tagged CDCs was as previously described [22,23].

### SDS-PAGE and Western blotting

Proteins were separated by electrophoresis in 10% (w/v) SDS-polyacrylamide gels and transferred to nitrocellulose [15]. Western blots were immunostained using rabbit anti-His-ALN (prepared by immunization of a rabbit with His-ALN, Antibodies Inc., Davis, CA) and rabbit anti-goat IgG(H+L)-peroxidase conjugate (KPL), as the primary and secondary antibodies, respectively. Rabbit antiserum against PFO was kindly provided by Rodney K. Tweten, University of Oklahoma Health Sciences Center, OK.

### Hemolytic assays

The hemolytic titers of His-ALN preparations were determined by incubation of two-fold serial dilutions of protein with an equal volume of 0.5% blood (Cleveland Scientific, Bath, OH) at 37°C for 1 h [14]. The hemolytic titer was the reciprocal of the highest dilution which resulted in 50% cell lysis, expressed as hemolytic units (HU) [14]. The specific activity of purified His-ALN was determined as HU/μg protein. Thiol activation was assessed by incubation of 5 HU His-ALN with 2% β-mercaptoethanol for 10 min at room temperature, prior to performing a hemolytic assay with human blood (Cleveland Scientific). Cholesterol inhibition was assessed by incubation of 5 HU His-ALN with 0.01-1 μM cholesterol for 30 min at room temperature with shaking, prior to performing a hemolytic assay with human blood. Cholesterol was diluted in absolute ethanol and an equal volume of ethanol was used as the cholesterol-free control. His-tagged perfringolysin O (PFO) [24] and His-tagged PLO [14] were used as controls in the various hemolytic assays. For some experiments hemolysis assays were performed as described [22,23].

### Epithelial cell cytotoxicity

The epithelial cell cytotoxicity of His-ALN was determined using the CellTiter 96^® ^Aqueous One Solution Cell Proliferation Assay (Promega). A549 (human lung, CCL-185), CHO (hamster ovary, CCL-61), HCT-8 (human colon, CCL-244), J774A.1 (mouse macrophage, TIB-67), MDBK (bovine kidney, CCL-22), MDCK (canine kidney, CCL-34) and RK-13 (rabbit kidney, CCL-37) cells were cultured in Iscove's Modified Dulbecco's Medium or RMPI 1640 with 10% fetal bovine serum and 10 μg/ml gentamicin in a humidified, 5% CO_2 _atmosphere at 37°C. Cells were seeded into 96-well plates at 2 × 10^4 ^cells/well and incubated for 18 h to achieve 80% confluence. Triplicate wells were incubated with doubling dilutions of His-ALN (0-2000 ng) and incubated for 2 h, prior to addition of substrate for 3 h. Determination of cell viability was performed using the appropriate control values (Promega).

### Membrane binding assay

The membrane binding assay was performed using erythrocytes as previously described [25]. His-ALN was diluted to 12.5 μg ml^-1 ^in PBS, 40 μl was added to an equal volume of 50% (v/v) blood and the mixture was incubated on ice for 20 min. Cells were harvested by centrifugation at 14,000 g for 5 min at 4°C, resuspended in SDS-PAGE sample buffer and subjected to SDS-PAGE and Western blotting with antiserum against His-ALN.

## Results

### Cloning and nucleotide sequence determination of *aln*

**Figure 1:**
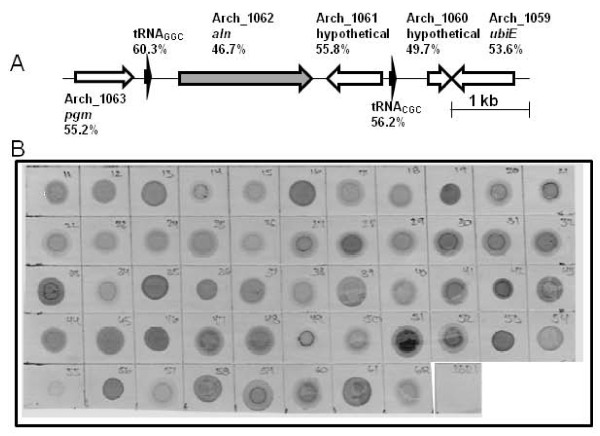
**Map of the *A. haemolyticum aln *region and presence of *aln *in clinical isolates**. (a) Map of the *aln *gene region of strain ATCC 9345 (= DSM20595 = 11018). The open arrows indicate the gene and the direction of transcription. Gene names are given and the number indicates the %G+C of the gene. A bar indicating 1 kb is shown on the right. (b) DNA dot hybridization of genomic DNA from *A. haemolyticum *strains with an *aln-*specific probe. Genomic DNA from 52 *A. haemolyticum *isolates and *T. pyogenes *BBR1, as a negative control (~500 ng each), was spotted onto a nylon membrane and hybridized with *aln-*specific probe under high stringency conditions. *A. haemolyticum *ATCC9345 DNA is in the second from last spot. *T. pyogenes *BBR1 DNA is in the last spot.

A draft genome sequence of *A. haemolyticum *ATCC 9345 was determined and consists of 46 contigs that encompass ~1.945 Mb in size (D. J. McGee, S. J. Billington, and B. J. Jost, unpublished). 1,639 ORFs were preliminarily identified using the Rapid Annotation using Subsystem Technology (RAST) Server [26]. Within this sequence, we identified ORF Arch_1062, the translation of which displayed similarity to other CDCs. The 1,710 bp gene was designated *aln*, for arcanolysin (ALN). Upstream of *aln *are a phosphoglycerate mutase gene (*pgm*; Arch_1063) (EC 5.4.2.1) and an alanine tRNA_GGC _(Figure 1). In the 426 bp intergenic region are regulatory signals predicted to be involved in *aln *transcription, including a putative σ^70 ^promoter and 3 direct repeats (ATTTT(G/C)(G/T)T) which are similar to those found immediately upstream of *plo*, encoding PLO, the CDC of *T. pyogenes *[27]. 6 bp downstream of *aln *is a transcriptional terminator with a ΔG = -18.05 kcal/mol. Downstream of *aln *and divergently transcribed is Arch_1061. The Arch_1061 protein displays amino acid similarity to hypothetical proteins from a number of genome sequences, including *Corynebacterium jeikeium *(GenBank YP_249820.1), and features a signal sequence. Further downstream is an additional alanine tRNA_CGC_, which is 91% identical at the nucleotide level to the alanine tRNA_GGC _upstream of *aln*. Further downstream of the 2^nd ^alanine tRNA is Arch_1060, a gene that is predicted to encode a conserved hypothetical protein related to *Corynebacterium diphtheriae *(DIP0761), and a gene, Arch_1059 (*ubiE*), with similarity to type II or SAM-dependent methyltransferases (EC 2.1.1.-).

The %G+C for *aln *is 46.7% (Figure 1) compared with 49.7-60.3% for the surrounding genes and 53.1% for the entire genome. Given the lower %G+C of the *aln *gene and the presence of flanking tRNA genes, which can act as sites of foreign gene insertion [28], it is possible that the *A. haemolyticum aln *gene was acquired by horizontal gene transfer.

### *aln *is widely distributed in *A. haemolyticum *isolates

The prevalence of the *aln *gene was determined by DNA hybridization. A DIG-labeled probe spanning bases 492-1,052 of the *aln *ORF was hybridized to genomic DNA from *A. haemolyticum *ATCC9345, 51 *A. haemolyticum *clinical isolates (Table 1) and *T. pyogenes *BBR1, as a negative control. The *aln *probe hybridized at high stringency to all *A. haemolyticum *isolates (n = 52), but not *T. pyogenes *genomic DNA (Figure 1b), indicating that this gene appears to be highly prevalent in *A. haemolyticum*. The region of *aln *from which the probe was derived has 62.8% identity to the corresponding nucleotide region in *plo *of *T. pyogenes*. Under high stringency hybridization conditions, DNA sequences which are less than 70% identical do not hybridize.

### Analysis of the primary structure of ALN

**Figure 2:**
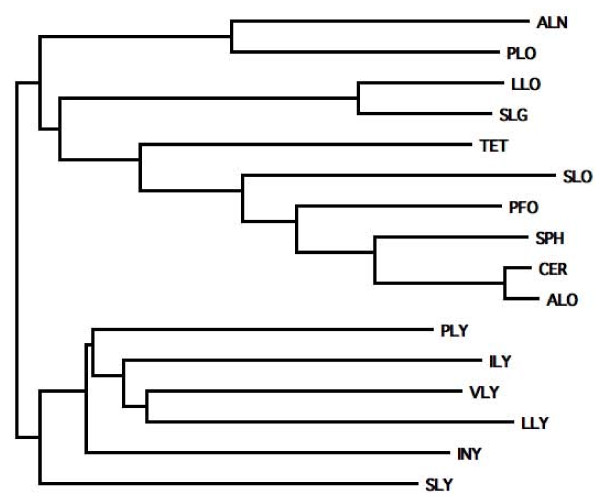
**Neighbor joining tree of amino acid sequences showing the relationship of ALN to other selected CDC family members**. Abbreviations and gi ascession numbers from the NCBI protein database: ALN, arcanolysin (259156857); PLO, pyolysin (2252800); LLO, listeriolysin O (16802248); SLG seeligeriolysin (40889013); TET, tetanolysin (28211522); SLO, streptolysin O (15674372); PFO, perfringolysin O (18309145); SPH, sphaericolysin (146455206); CER, cereolysin (62550724); ALO, anthrolysin O (49186114); PLY, pneumolysin (15901747); ILY, intermedilysin (6729344); VLY, vaginolysin (187940699); LLY, lectinolysin (190576835); INY, inerolysin (259167149); SLY, suilysin (253752120).

**Figure 3:**
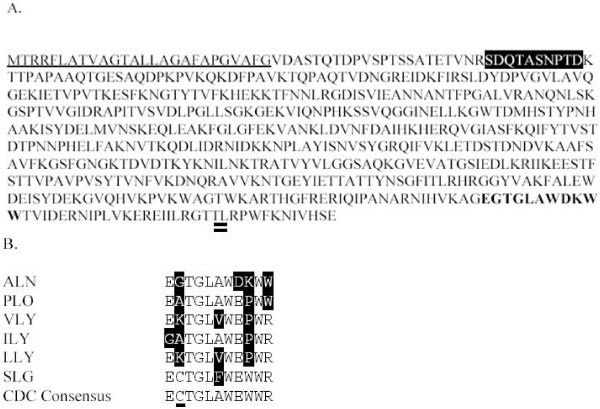
**Salient features of the ALN predicted amino acid sequence**. (a) ALN sequence with predicted signal sequence (underlined), putative PEST motif (inverse), undecapeptide (bold), and cholesterol-interacting TL motif (double underlined). (b) Undecapeptide sequences of ALN, other CDC undecapeptides known to differ from consensus, and the consensus CDC undecapeptide. The cysteine conserved in thiol-activated CDCs (but absent from ALN) is underlined in the consensus sequence. Differences from consensus depicted as inverse letters. Abbreviations as in Figure 2.

The predicted ALN protein is 569 amino acids in length, including a 26 amino acid signal sequence predicted by SignalP. The mature protein lacking the signal sequence has a predicted molecular mass of 60.1 kDa. ALN is most similar to PLO with 59.4% and 71.5% amino acid identity and similarity (Figure 2) and has ~50% similarity to other CDC family members. Within the ALN N-terminus, the pestfind algorithm identified a putative PEST sequence not present in PLO or most other CDC sequences (Figure 3a). Listeriolysin O (LLO), which contains a *bona fide *PEST sequence [29], returned a pestfind score of 4.71, while ALN had a score of 7.58, indicating a higher probability of containing a functional PEST sequence. Given that *A. haemolyticum *invades host cells [9], it is possible that the PEST sequence allows for a similar compartmentalization of ALN activity within the host cell. Like PLO, the predicted amino acid sequence of ALN has a variant undecapeptide in domain 4 and both lack the conserved cysteine residue (Figure 3b). The tryptophan spacing of ALN and PLO (WxxWW) also differs from the consensus sequence (WxWW) (Figure 3b).

### Cloning and expression of His-ALN

**Figure 4:**
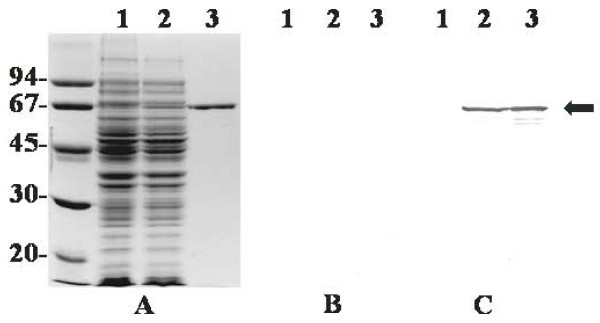
**Overexpression and purification of His-ALN**. Whole-cell lysates of IPTG-induced cultures of DH5αMCR(pTrcHis B) (lane 1) and DH5αMCR (pBJ51) (lane 2) and 500 ng purified His-ALN (lane 3) were subjected to SDS-PAGE. Separated proteins were stained with Coomassie brilliant blue (a) or were transferred to nitrocellulose by Western blotting and immunostained with 1/5000 rabbit pre-immune serum (b) or rabbit anti-His-ALN (c). The position of the ~64 kDa His-ALN band is indicated by the arrow. Molecular mass markers (kDa) are indicated on the left.

SDS-PAGE and Coomassie Brilliant Blue staining of IPTG-induced cultures of pBJ51-containing *E. coli *indicated the presence of an over-expressed protein of ~64 kDa (Figure 4a). His-ALN was purified to > 95% homogeneity using TALON resin (Figure 4a), and the size of this protein (~64 kDa) corresponded to its predicted molecular mass. Antiserum against ALN, but not pre-immune antiserum, reacted specifically with His-ALN and some possible HIS-ALN degradation products (Figure 4b and 4c).

### Recombinant ALN has cytotoxic activity

**Figure 5:**
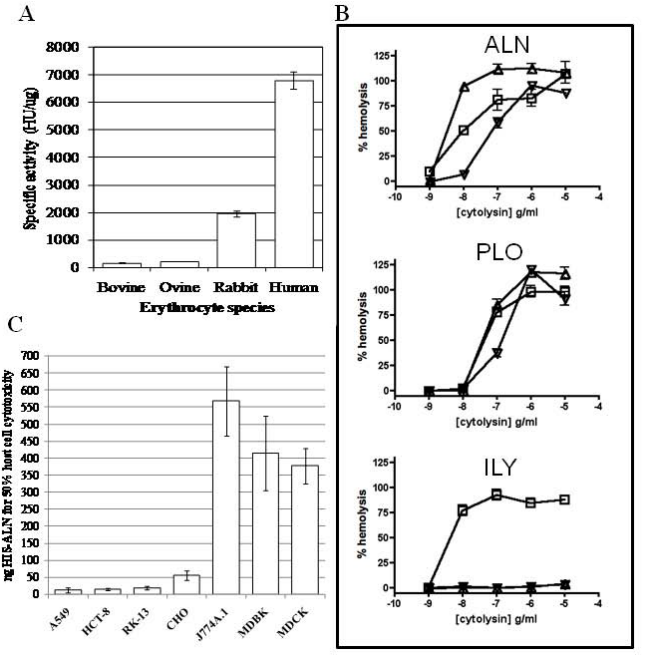
**ALN has differential activity on cells from various mammalian species**. (a) The specific activities of ALN were determined by incubation of dilutions of His-ALN with erythrocytes from different host species. Results are an average of at least three independent experiments conducted in duplicated and error bars represent standard deviation. (b) The species selectivity of ALN was compared to ILY and PLO in hemolysis assays using human (square), horse (triangle), and pig (inverted triangle) erythrocytes. Representative of two experiments conducted in triplicate and error bars represent standard error of the mean. (c) Dilutions of His-ALN were added to cultured host cells and the amount of ALN required to reduce the cell viability by 50% was determined using the CellTiter 96^® ^Aqueous One Solution Cell Proliferation Assay (Promega). Error bars indicate one standard deviation from the mean calculated from the averages of at least three independent experiments conducted in triplicate.

*A. haemolyticum *is not strongly hemolytic when grown on ovine (sheep) blood agar [10]. Likewise, the *E. coli *strain expressing His-ALN did not display hemolysis when grown on bovine blood agar (data not shown). Similarly, His-ALN displays low hemolysis with bovine or ovine erythrocytes (Figure 5a). In contrast, His-ALN had ~4- and 10-fold increased hemolytic activity on rabbit and human erythrocytes, respectively (Figure 5a). This is in contrast to PFO or PLO, which show little difference in specific activity on erythrocytes from different hosts. Consistent with these findings, hemolysis assays demonstrated that ALN has a preference for horse or human cells over porcine cells but lyses all of these at high toxin concentrations (Figure 5b). This is in contrast to intermedilysin (ILY) from *Streptococcus intermedius*, which retains human-specific tropism over a wide concentration range, and PLO, which is less selective than ALN (Figure 5b).

The highly-conserved Cys residue in the undecapeptide of CDCs is responsible for Thiol activation of this group of toxins [30]. ALN lacks the Cys residue in the undecapeptide (Figure 3a), and like PLO [14], its activity was unaffected by treatment with β-mercaptoethanol (data not shown).

We also determined the effect of recombinant ALN on cultured mammalian cells. His-ALN was applied to human, bovine, canine, hamster, mouse and rabbit cell lines and was highly active on human and rabbit cells (Figure 5c), with low activity on bovine, mouse and canine cells. This toxin had intermediate activity on hamster cells (Figure 5c). This finding mirrors the activity of ALN on blood from different host species (Figure 5a), and is less species-specific than intermedilysin (ILY) or vaginolysin (VLY) [23,31]. ILY, VLY, and lectinolysin (LLY) use human CD59 (hCD59) as a membrane receptor [23,32,33], leading to host-specificity. Unlike these other CDC toxins ALN hemolysis was not blocked with a monoclonal antibody against hCD59 (data not shown). Consistent with this finding, the predicted ALN amino acid sequence lacks the Tyr-X-Tyr-X_14_-Ser-Arg signature motif common to all known hCD59-dependent CDCs [33].

### The activity of ALN is less sensitive to cholesterol inhibition than PFO

**Figure 6:**
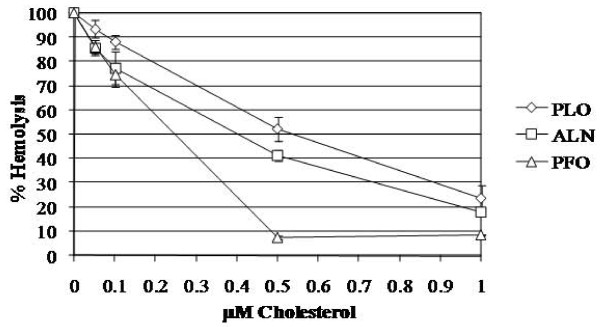
**ALN, a cholesterol-dependent cytolysin, has hemolytic activity that is less sensitive to cholesterol inhibition than PFO**. His-tagged CDCs were preincubated with dilutions of cholesterol for 30 min at room temperature prior to hemolytic assay. Abbreviations as in Figure 2. Error bars indicate one standard deviation from the mean calculated from the averages of three independent experiments conducted in triplicate.

Given the more restrictive host species preference of ALN over that of PFO, along with the variant undecapeptide sequence in ALN, we hypothesized that ALN might be less sensitive to inhibition by free cholesterol. As expected, PFO activity was almost completely inhibited by exogenous 0.5 μM cholesterol (7.6%; Figure 6). In contrast, PLO and ALN retained 52.5% and 41.4% activity, respectively, when incubated with 0.5 μM cholesterol and retained ~20% of hemolytic activity at 1 μM cholesterol (Figure 6). These data indicate that ALN and PLO have intermediate sensitivity to cholesterol compared to a CDC (PFO) with the conserved undecapeptide sequence.

### ALN binds differentially to host cell membranes

**Figure 7:**
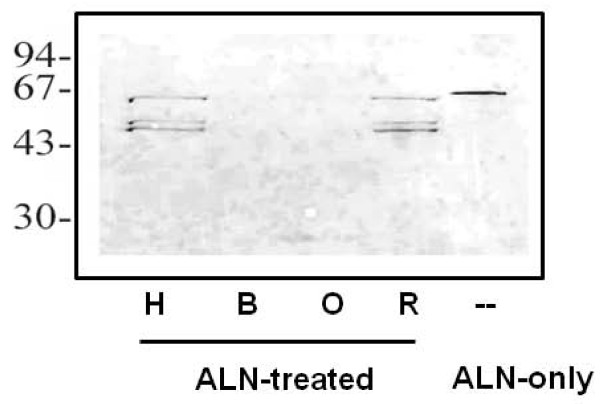
**ALN has a differential ability to bind to erythrocyte cell membranes from different host species**. His-ALN (500 ng) or buffer (negative control) was added to erythrocytes, and the mixture was incubated on ice for 20 min. Untreated (no reactivity, data not shown) or ALN-treated erythrocyte membrane fractions from human (H), bovine (B), ovine (O) or rabbit (R) blood were separated by SDS-PAGE, transferred to nitrocellulose, and immunostained with 1/1000 rabbit anti-His-ALN. His-Aln (500 ng) in absence of erythrocyte membrane fractions (ALN) serves as the positive control. Molecular mass markers (kDa) are indicated on the left.

Hemolytic assays measure the full spectrum of CDC binding, oligomerization and pore formation leading to cell lysis. However, initial toxin binding to membranes can be determined by incubation of CDCs with host cells at 4°C, which prevents subsequent oligomerization and pore formation [34]. Using this approach, His-ALN bound to human and rabbit erythrocytes as determined by Western blotting (Figure 7). Probable ALN degradation products were also detected. His-ALN did not exhibit detectable binding to bovine or ovine erythrocyte membranes under these conditions. As a control, His-PFO was incubated with human, bovine, ovine or rabbit erythrocytes, and bound toxin was detected with anti-PFO antiserum. His-PFO bound to all cell types at approximately equivalent amounts (data not shown). These data suggest that ALN host preference may occur at the initial contact of the toxin with the host cell membrane.

## Discussion

The CDCs are a family of bacterial toxins produced by diverse Gram-positive bacteria and are generally important in pathogenesis [35-37]. CDCs have a four-domain structure and a conserved C-terminal undecapeptide sequence in domain 4 that is important for toxin function. Soluble CDC monomers bind to host membrane targets, oligomerize into a large homomeric structure known as the prepore complex, and transition to a true pore, leading to cytolysis of target cells [38]. CDCs interact with membrane cholesterol through a conserved threonine-leucine pair in domain 4, and this interaction is crucial to the formation of functional pores [39]. Some CDCs, including ILY, VLY, and LLY, require the presence of hCD59 as a membrane receptor, conferring human-specific activity [23,33,40]. Among the CDCs, PLO is unusual, as it contains a variant of the highly conserved domain 4 undecapeptide, and this divergent sequence is essential for full PLO activity [41]. The ALN undecapeptide is most similar to that of PLO (Figure 3B), in that it retains the three tryptophan residues of the consensus undecapeptide but employs an alternate spacing (i.e. WxxWW rather than WxWW). The tryptophan residues of the undecapeptide are known to be important for insertion of domain 4 into host cell membranes [42]. Like the human-specific CDCs (VLY, ILY, and LLY), ALN contains a proline in its undecapeptide sequence. However, the hemolytic activity of ALN was not blocked by antibodies to human CD59, which acts as a receptor for the human-specific CDCs [23,32,33], suggesting that ALN may interact with a distinct membrane receptor, perhaps in addition to cholesterol. The nature of the ALN receptor is currently unknown and is under investigation. Although the cysteine residue in the consensus undecapeptide confers the property of thiol activation to CDCs, the cysteine is not essential for streptolysin O and pneumolysin toxin function [43,44]. The human-specific CDCs (VLY, ILY, LLY), PLO, and ALN all lack this conserved cysteine residue, but the contribution of this sequence variation to toxin function is not yet known for these toxins.

Some CDCs have a number of functions beyond simple pore formation. *Streptococcus pyogenes *uses streptolysin O to introduce a bacterial effector into host cells via a novel mechanism termed cytolysin-mediated translocation (CMT) [45]. At sublytic concentrations, CDCs may act as ligands for toll-like receptors [46,47] and may induce a cycle of p38 mitogen-activated protein kinase (MAPK) phosphorylation and dephosphorylation [48,49]. LLO allows *Listeria monocytogenes *to escape from the vacuole into the cytoplasm where the organism can rapidly multiply [50]. The site-specific nature of LLO is controlled by cytosolic down-regulation of LLO function due to an N-terminal PEST-like sequence, which usually targets eukaryotic proteins for cytosolic degradation. The PEST sequence results in a substantially reduced half-life of LLO in the cytoplasm of the host cell [29].

## Conclusions

ALN has several unique features among the CDC family. ALN has a variant undecapeptide and possesses an unusual N-terminal extension, with a putative PEST sequence. Moreover, ALN lacks the conserved cysteine of thiol-activated CDCs, explaining why β-mercaptothanol had no effect on ALN function. The unique sequences and predicted structural features of ALN will make it an interesting toxin to conduct future structure-function analyses to identify additional unique properties of this toxin. ALN displays an unusual pattern of target cell species selectivity, with high activity against human, horse, and rabbit cells and lesser activity against cells derived from other species. This selectivity appears to function at the level of membrane binding and may contribute to the host range of *A. haemolyticum*. Further work will focus on understanding the role of ALN in *A. haemolyticum *pathogenesis.

## Competing interests

The authors declare that they have no competing interests.

## Authors' contributions

BHJ, EAL and AJR designed and conducted the experiments and analyzed data, BHJ drafted the manuscript, AJR, SJB and DJM revised the manuscript and figures. All authors read and approved the final manuscript.

## References

[B1] Linder R *Rhodococcus equi *and *Arcanobacterium haemolyticum*: two "coryneform" bacteria increasingly recognized as agents of human infection Emerging Infectious Diseases 1997 3 145 153 10.3201/eid0302.970207 9204295 PMC2627624

[B2] Banck G Nyman M Tonsillitis and rash associated with *Corynebacterium haemolyticum* J Infect Dis 1986 154 1037 1040 10.1093/infdis/154.6.1037 3782868

[B3] Mackenzie A Fuite LA Chan FT King J Allen U MacDonald N Diaz-Mitoma F Incidence and pathogenicity of *Arcanobacterium haemolyticum *during a 2-year study in Ottawa Clin Infect Dis 1995 21 177 181 10.1093/clinids/21.1.177 7578727

[B4] Miller RA Brancato F Holmes KK *Corynebacterium haemolyticum *as a cause of pharyngitis and scarlatiniform rash in young adults Ann Intern Med 1986 105 867 872 3535603 10.7326/0003-4819-105-6-867

[B5] Collins MD Jones D Schofield GM Reclassification of '*Corynebacterium haemolyticum*' (MacLean, Liebow & Rosenberg) in the genus *Arcanobacterium *gen. nov. as *Arcanobacterium haemolyticum *nom. rev., comb. nov J Gen Microbiol 1982 128 1279 1281 7119737 10.1099/00221287-128-6-1279

[B6] Jost BH Billington SJ *Arcanobacterium pyogenes*: molecular pathogenesis of an animal opportunist Antonie van Leeuwenhoek 2005 88 87 102 10.1007/s10482-005-2316-5 16096685

[B7] Cuevas WA Songer JG *Arcanobacterium haemolyticum *phospholipase D is genetically and functionally similar to *Corynebacterium pseudotuberculosis *phospholipase D Infect Immun 1993 61 4310 4316 8406819 10.1128/iai.61.10.4310-4316.1993 PMC281159

[B8] Soucek A Souckova A Toxicity of bacterial sphingomyelinases D J Hyg Epidemiol Microbiol Immunol 1974 18 327 335 4213711

[B9] Lucas EA Billington SJ Carlson P McGee DJ Jost BH Phospholipase D promotes *Arcanobacterium haemolyticum *adhesion via lipid raft remodeling and host cell death following bacterial invasion BMC Microbiology 2010 10 270 10.1186/1471-2180-10-270 20973961 PMC2978216

[B10] Funke G von Graevenitz A Clarridge III JE Bernard KA Clinical microbiology of coryneform bacteria Clin Microbiol Rev 1997 10 125 159 8993861 10.1128/cmr.10.1.125 PMC172946

[B11] Hassan AA Ulbegi-Mohyla H Kanbar T Alber J Lammler C Abdulmawjood A Zschock M Weiss R Phenotypic and genotypic characterization of *Arcanobacterium haemolyticum *isolates from infections of horses Journal of Clinical Microbiology 2009 47 1 124 128 10.1128/JCM.01933-08 19020059 PMC2620868

[B12] MacLean PD Liebow AA Rosenberg AA A haemolytic bacterium resembling *Corynebacterium ovis *and *Corynebacterium pyogenes *in man J Infect Dis 1946 79 69 90 10.1093/infdis/79.1.69 20996930

[B13] Linder R Bernheimer AW Enzymatic oxidation of membrane cholesterol in relation to lysis of sheep erythrocytes by corynebacterial enzymes Arch Biochem Biophys 1982 213 395 404 10.1016/0003-9861(82)90565-3 7073283

[B14] Billington SJ Jost BH Cuevas WA Bright KR Songer JG The *Arcanobacterium *(*Actinomyces*) *pyogenes *hemolysin, pyolysin, is a novel member of the thiol-activated cytolysin family J Bacteriol 1997 179 6100 6106 9324258 10.1128/jb.179.19.6100-6106.1997 PMC179514

[B15] Ausubel FM Brent R Kingston RE Moore DD Seidman JG Smith JA Struhl K Current protocols in molecular biology 1994 1 New York, NY: Greene Publishing Associates and John Wiley and Sons, Inc.

[B16] Yasawong M Teshima H Lapidus A Nolan M Lucas S Glavina Del Rio T Tice H Cheng JF Bruce D Detter C Complete genome sequence of *Arcanobacterium haemolyticum *type strain (11018) Stand Genomic Sci 2010 3 2 126 135 10.4056/sigs.1123072 21304742 PMC3035375

[B17] Altschul SF Madden TL Schäffer AA Zhang J Zhang Z Miller W Lipman DJ Gapped BLAST and PSI-BLAST: a new generation of protein database search programs Nucleic Acids Res 1997 25 3389 3402 10.1093/nar/25.17.3389 9254694 PMC146917

[B18] Lowe TM Eddy SR tRNAscan-SE: a program for improved detection of transfer RNA genes in genomic sequence Nucl Acids Res 1997 25 955 964 10.1093/nar/25.5.955 9023104 PMC146525

[B19] Nielsen H Engelbrecht J Brunak S von Heijne G Identification of prokaryotic and eukaryotic signal peptides and prediction of their cleavage sites Protein Eng 1997 10 1 6 10.1093/protein/10.1.1 9051728

[B20] Zucker M Mfold web server for nucleic acid folding and hybridization prediction Nucl Acids Res 2003 31 3406 3415 10.1093/nar/gkg595 12824337 PMC169194

[B21] Thompson JD Higgins DG Gibson TJ CLUSTAL W: improving the sensitivity of progressive multiple sequence alignment through sequence weighting, position-specific gap penalties and weight matrix choice Nucleic Acids Res 1994 22 4673 4680 10.1093/nar/22.22.4673 7984417 PMC308517

[B22] Rampersaud R Planet PJ Randis TM Kulkarni R Aguilar JL Lehrer RI Ratner AJ Inerolysin, a cholesterol-dependent cytolysin produced by *Lactobacillus iners* Journal of Bacteriology 2011 193 5 1034 1041 10.1128/JB.00694-10 21169489 PMC3067590

[B23] Gelber SE Aguilar JL Lewis KL Ratner AJ Functional and phylogenetic characterization of Vaginolysin, the human-specific cytolysin from *Gardnerella vaginalis* Journal of Bacteriology 2008 190 11 3896 3903 10.1128/JB.01965-07 18390664 PMC2395025

[B24] Fernandez-Miyakawa ME Jost BH Billington SJ Uzal FA Lethal effects of *Clostridium perfringens *epsilon toxin are potentiated by alpha and perfringolysin-O toxins in a mouse model Vet Microbiol 2007 127 379 385 17997054 10.1016/j.vetmic.2007.09.013 PMC2276457

[B25] Jost BH Trinh HT Songer JG Billington SJ Immunization with genetic toxoids of the *Arcanobacterium pyogenes *cholesterol-dependent cytolysin, pyolysin, protects mice against infection Infect Immun 2003 71 2966 2969 10.1128/IAI.71.5.2966-2969.2003 12704180 PMC153263

[B26] Meyer F Paarmann D D'Souza M Olson RD Glass EM Kubal M Paczian T Rodriguez A Stevens R Wilke A The metagenomics RAST server - a public resource for the automatic phylogenetic and functional analysis of metagenomes BMC Bioinformatics 2008 9 386 10.1186/1471-2105-9-386 18803844 PMC2563014

[B27] Rudnick ST Jost BH Songer JG Billington SJ The gene encoding pyolysin, the pore-forming toxin of *Arcanobacterium pyogenes*, resides within a genomic islet flanked by essential genes FEMS Microbiol Lett 2003 225 241 247 10.1016/S0378-1097(03)00527-5 12951248

[B28] Williams KP Integration sites for genetic elements in prokaryotic tRNA and tmRNA genes: sublocation preference of integrase subfamilies Nucl Acids Res 2002 30 866 875 10.1093/nar/30.4.866 11842097 PMC100330

[B29] Decatur AL Portnoy DA A PEST-like sequence in listeriolysin O essential for *Listeria monocytogenes *pathogenicity Science 2000 290 992 995 10.1126/science.290.5493.992 11062133

[B30] Alouf JE Billington SJ Jost BH Alouf JE, Popoff MR Repertoire and general features of the family of cholesterol-dependent cytolysins The comprehensive sourcebook of bacterial protein toxins 2006 3 London: Academic Press 643 658

[B31] Nagamune H Streptococcal cytolysins Seikagaku 1997 69 343 348 9214847

[B32] Giddings KS Zhao J Sims PJ Tweten RK Human CD59 is a receptor for the cholesterol-dependent cytolysin intermedilysin Nat Struct Mol Biol 2004 11 1173 1178 10.1038/nsmb862 15543155

[B33] Wickham SE Hotze EM Farrand AJ Polekhina G Nero TL Tomlinson S Parker MW Tweten RK Mapping the Intermedilysin-Human CD59 Receptor Interface Reveals a Deep Correspondence with the Binding Site on CD59 for Complement Binding Proteins C8{alpha} and C9 J Biol Chem 2011 286 23 20952 20962 10.1074/jbc.M111.237446 21507937 PMC3121471

[B34] de los Toyos JR Mendez FJ Aparicio JF Vázquez F del Mar García Suárez M Fleites A Hardisson C Morgan PJ Andrew PW Mitchell TJ Functional analysis of pneumolysin by use of monoclonal antibodies Infect Immun 1996 64 480 484 8550195 10.1128/iai.64.2.480-484.1996 PMC173789

[B35] Gilbert RJ Cholesterol-dependent cytolysins Advances in Experimental Medicine & Biology 2010 677 56 66 10.1007/978-1-4419-6327-7_5 20687480

[B36] Heuck AP Moe PC Johnson BB The cholesterol-dependent cytolysin family of gram-positive bacterial toxins Sub-Cellular Biochemistry 2010 51 551 577 10.1007/978-90-481-8622-8_20 20213558

[B37] Tweten R Cholesterol-dependent cytolysins, a family of versatile pore-forming toxins Infect Immun 2005 73 6199 6209 10.1128/IAI.73.10.6199-6209.2005 16177291 PMC1230961

[B38] Heuck AP Tweten RK Johnson AE Assembly and topography of the prepore complex in cholesterol-dependent cytolysins J Biol Chem 2003 278 31218 31225 10.1074/jbc.M303151200 12777381

[B39] Farrand AJ LaChapelle S Hotze EM Johnson AE Tweten RK Only two amino acids are essential for cytolytic toxin recognition of cholesterol at the membrane surface Proceedings of the National Academy of Sciences of the United States of America 2010 107 9 4341 4346 10.1073/pnas.0911581107 20145114 PMC2840085

[B40] Giddings KS Johnson AE Tweten RK Redefining cholesterol's role in the mechanism of the cholesterol-dependent cytolysins Proc Natl Acad Sci USA 2003 100 11315 11320 10.1073/pnas.2033520100 14500900 PMC208754

[B41] Billington SJ Songer JG Jost BH The variant undecapeptide sequence of the *Arcanobacterium pyogenes *haemolysin, pyolysin, is required for full cytolytic activity Microbiology 2002 148 3947 3954 12480898 10.1099/00221287-148-12-3947

[B42] Soltani CE Hotze EM Johnson AE Tweten RK Specific protein-membrane contacts are required for prepore and pore assembly by a cholesterol-dependent cytolysin Journal of Biological Chemistry 2007 282 21 15709 15716 10.1074/jbc.M701173200 17412689 PMC3746338

[B43] Pinkney M Beachey E Kehoe M The thiol-activated toxin streptolysin O does not require a thiol group for cytolytic activity Infect Immun 1989 57 2553 2558 2663727 10.1128/iai.57.8.2553-2558.1989 PMC313485

[B44] Saunders FK Mitchell TJ Walker JA Andrew PW Boulnois GJ Pneumolysin, the thiol-activated toxin of *Streptococcus pneumoniae*, does not require a thiol group for in vitro activity Infect Immun 1989 57 2547 2552 2744861 10.1128/iai.57.8.2547-2552.1989 PMC313484

[B45] Madden JC Ruiz N Caparon M Cytolysin-mediated translocation (CMT): a functional equivalent of type III secretion in Gram-positive bacteria Cell 2001 104 143 152 10.1016/S0092-8674(01)00198-2 11163247

[B46] Malley R Henneke P Morse SC Cieslewicz MJ Lipsitch M Thompson CM Kurt-Jones E Paton JC Wessels MR Golenbock DT Recognition of pneumolysin by Toll-like receptor 4 confers resistance to pneumococcal infection Proceedings of the National Academy of Sciences of the United States of America 2003 100 4 1966 1971 10.1073/pnas.0435928100 12569171 PMC149942

[B47] Park JM Ng VH Maeda S Rest RF Karin M Anthrolysin O and other gram-positive cytolysins are toll-like receptor 4 agonists J Exp Med 2004 200 1647 1655 10.1084/jem.20041215 15611291 PMC2211988

[B48] Aguilar JL Kulkarni R Randis TM Soman S Kikuchi A Yin Y Ratner AJ Phosphatase-dependent regulation of epithelial mitogen-activated protein kinase responses to toxin-induced membrane pores PLoS ONE [Electronic Resource] 2009 4 11 e8076 10.1371/journal.pone.0008076 PMC2778951 19956644

[B49] Ratner AJ Hippe KR Aguilar JL Bender MH Nelson AL Weiser JN Epithelial cells are sensitive detectors of bacterial pore-forming toxins Journal of Biological Chemistry 2006 281 18 12994 12998 10.1074/jbc.M511431200 16520379 PMC1586115

[B50] Vazquez-Boland JA Kuhn M Berche P Chakraborty T Dominguez-Bernal G Goebel W Gonzalez-Zorn B Wehland J Kreft J *Listeria *pathogenesis and molecular virulence determinants Clin Microbiol Rev 2001 14 584 640 10.1128/CMR.14.3.584-640.2001 11432815 PMC88991

